# Draft Genome Sequence of *Cylindrospermopsis* sp. Strain CR12 Extracted from the Minimetagenome of a Nonaxenic Unialgal Culture from a Tropical Freshwater Lake

**DOI:** 10.1128/genomeA.01726-15

**Published:** 2016-02-11

**Authors:** Nur Hazimah Mohamed Nor, Boon Fei Tan, Shu Harn Te, Janelle R. Thompson, Karina Yew-Hoong Gin

**Affiliations:** a NUS Environmental Research Institute, National University of Singapore, Singapore; b Centre for Environmental Sensing and Modelling, Singapore-MIT Alliance for Research and Technology, Singapore; c Department of Civil and Environmental Engineering, National University of Singapore, Singapore

## Abstract

*Cylindrospermopsis* is known to be one of the major bloom-forming cyanobacterial genera in many freshwater environments. We report here the draft genome sequence of a tropical *Cylindrospermopsis* sp. strain, CR12, which is capable of producing the hepatotoxic cylindrospermopsin.

## GENOME ANNOUNCEMENT

*Cylindrospermopsis* has been identified as one of the major cyanobacterial bloom members in many eutrophic lakes in tropical and temperate climate zones (1). Some species are nontoxigenic, while several others are capable of producing the hepatotoxic cylindrospermopsin (2) and neurotoxic saxitoxin (paralytic shellfish poison), which can be life-threatening if ingested (3). Additional *Cylindrospermopsis* genomes from local freshwater ecosystems can be valuable for the identification of genes encoding secondary metabolites that may have an impact on water quality.

Two nonaxenic cultures (CR1 and CR2) containing a dominant cyanobacterium resembling the morphology of *Cylindrospermopsis* were isolated from a tropical freshwater lake using a micropipetting method (4) performed under a microscope. An individual filament was picked and transferred repeatedly in sterile water droplets to eliminate background protists before culturing in growth medium (MLA medium) (5).

Total DNA was isolated from the two cultures using a PowerWater DNA isolation kit (Mo Bio). A sequencing library was separately constructed for the DNA isolated from each culture, using the Illumina TruSeq DNA Nano kit, and sequenced with Illumina HiSeq 2000 employing the 2 × 250-bp paired-end protocol. Quality controls, including barcode and adaptor sequence removal, followed by *de novo* assembly, were conducted using CLC Genomics Workbench version 8 (CLC bio, USA). The contigs for *Cylindrospermopsis* in each of the two metagenomes were separated from those of other cooccurring heterotrophic bacteria using G+C content and contig coverage, as previously reported (6). The lack of a sequence contaminant was further confirmed using the best BLAST hits and principal component analysis of the tetranucleotide frequencies. Following this, the two *Cylindrospermopsis* draft genomes (~3.8 Mbp each) obtained from separate cultures were compared for their average nucleotide identity (ANI) (http://enve-omics.ce.gatech.edu/ani) (200-bp step size and 1,000-bp reading windows), revealing a 100% pairwise similarity (17,751 fragments), supported by 100% BLASTN identity in their 16S rRNA genes. Due to this similarity, the quality-controlled reads from the two draft genomes were merged and assembled as a single metagenome using the approach detailed above. Single-copy gene analyses (6) revealed that this genome is almost complete, and the two cultures were classified as the same strain, *Cylindrospermopsis* sp. CR12.

The 16S rRNA gene of CR12 is 99% similar to other *Cylindrospermopsis raciborskii* strains, supported by phylogenetic placement within the *C. raciborskii* clade and pairwise average nucleotide identity of 99% to another genome of *C. raciborskii* (accession no. NZ_ACYA00000000.1). The draft genome is 3.7 Mbp, contained in 136 scaffolds of 172 contigs (1,058 to 279,631 bp), with 40.1% G+C content, similar to other *C. raciborskii* strains, which have a genome size of 3.9 to 4.1 Mbp and 41% G+C content (7). Annotation using the RAST pipeline (8) predicted 3,479 protein-coding genes distributed across 356 SEED subsystem categories.

A complete cylindrospermopsin biosynthesis gene cluster was detected in the CR12 genome (e.g., CyrA, locus_tag ASL19_096), although a gene encoding saxitoxin analogues found in *Cylindrospermopsis* strains isolated in Brazil (3) was not detected in CR12 or in the genome of another *C. raciborskii* strain (accession no. NZ_ACYA00000000.1). As cylindrospermopsins can reduce the safety of local drinking water, this genome will serve as a reference for the further investigation of toxin production under different bloom conditions.

### Nucleotide sequence accession numbers.

This whole-genome shotgun project has been deposited at DDBJ/EMBL/GenBank under the accession no. LMVE00000000. The version described in this paper is version LMVE01000000.
